# Seepage Time Soft Sensor Model of Nonwoven Fabric Based on the Extreme Learning Machine Integrating Monte Carlo

**DOI:** 10.3390/s21072377

**Published:** 2021-03-29

**Authors:** Jing Zhang, Yiqiang Fan, Lulu Zhang, Chi Xu, Xiaobin Dong, Luyao Liu, Zhongping Zhang, Xianbo Qiu

**Affiliations:** 1 School of Information Science and Technology, Beijing University of Chemical Technology, Beijing 100029, China; jingzh@mail.buct.edu.cn (J.Z.); llzhang@mail.buct.edu.cn (L.Z.); 2016400134@mail.buct.edu.cn (C.X.); 18138439756@163.com (X.D.); 2019400156@mail.buct.edu.cn (L.L.); 2020400175@buct.edu.cn (Z.Z.); 2 School of Mechanical and Electrical Engineering, Beijing University of Chemical Technology, Beijing 100029, China; fanyq@mail.buct.edu.cn

**Keywords:** nonwoven fabric, porous media, MCELM, dyeing time

## Abstract

Nonwoven fiber materials are materials with multifunctional purposes, and are widely used to make masks for preventing the new Coronavirus Disease 2019. Because of the complexity and particularity of their structure, it becomes difficult to model the penetration and flow characteristics of liquid in nonwoven fiber materials. In this paper, a novel seepage time soft sensor model of nonwoven fabric, based on Monte Carlo (MC), integrating extreme learning machine (ELM) (MCELM) is proposed. The Monte Carlo method is used to expand data samples. Then, an ELM method is used to establish the prediction model of the dyeing time of the nonwoven fiber material overlaps with the porous medium, as well as the insertion degree and height of the different quantity of hides. Compared with the back propagation (BP) neural network and radial basis function (RBF) neural network, the results show that the prediction model based on the MCELM method has significant power in terms of accuracy and prediction speed, which is conducive to the precise and rapid manufacture of nonwoven fiber materials in practical applications between liquid seepage characteristics and structural characteristics of porous media. Furthermore, the relationship between the proposed models has certain value for predicting the behavior and use of nonwoven fiber materials with different structural characteristics and related research processes.

## 1. Introduction

Due to the impact of the new Coronavirus Disease 2019 (COVID-19), the public has a great need for masks, which are mainly used to prevent COVID-19. Nonwoven fabric, also known as nonwoven fabric, is the main raw material for masks, and is composed of oriented or random fibers. The porous media nonwoven fabric made of fibers has the characteristics of economy and environmental protection, as well as being soft and breathable, and has been widely used in industrial processes and daily life. In recent years, the nonwoven fabric industry has developed rapidly in China, and China has become the world’s largest nonwoven fabric producer and consumer. According to data from the China Industrial Textile Industry Association, nonwoven fabric production in China reached 6.213 million tons in 2019. The main application areas of nonwoven fabric include medical care, personal care, home life, and other fields [1,2]. However, the simulation prediction of nonwoven fabric impregnation time takes too long due to the complex production process. Therefore, an accurate prediction of impregnation time is conducive to fabric production and efficiency increases.

Corresponding to the extensive application market of nonwoven fabrics, the production process and technology of them have also been continuously developed and improved. Since the 1980s, nonwoven fabric industry in China has entered a continuous and rapid development stage. Although nonwoven fabric materials have been applied extensively in different fields, there are relatively few studies on liquid penetration and capillary flow in nonwovens. By repeatedly performing simulation calculations on a 3D image or digital geometric model of the material microstructure, and using the model to virtually change the geometry and redetermine the material properties, potential suitable materials can be found [3]. However, it is difficult to model and numerically analyze the microstructure of materials, such as nonwoven fabrics, in the actual application process, and the ideal geometric model does not completely match the real physical structure characteristics of them. Although studies have shown that the saturation of the amount of liquid absorbed by the capillary in nonwoven fabric varies considerably with the type of nonwoven fabric, and generally decreases with the increase in thickness of the nonwoven fabric [4], modeling and numerical analysis cannot really establish the relationship between the liquid penetration and capillary absorption capacity of nonwoven materials and structural characteristics. Based on the wide use of nonwoven fiber materials, a variety of performance requirements and restrictions, and the need for dyeing, are put forward for them. The need for dyeing also depends on the structural characteristics of the fiber material, to a certain extent. How to select the appropriate dyeing time according to the structural characteristics of the nonwoven fiber material is very important. This paper uses machine learning methods to predict the soaking time of nonwoven fabrics, and uses physical properties, such as aperture, to predict the penetration time of them. Macroscopic properties, such as porosity, have a direct impact on predicting the behavior and use of these materials.

This paper puts forward a novel extreme learning machine (ELM) method, combined with the Monte Carlo (MC) algorithm (MCELM), for analyzing and predicting the time required for the dyeing process. The real data of the dyeing industry is obtained by an experimental test, the sample size of which is insufficient. Then, the random simulation method based on the MC is adopted for expanding the original data. In order to prove that the expanded data can be used as experimental data, the hypothesis test method of Student’s *t* test (*T*-test) is employed to verify that there is no significant difference between the expanded data and the original data. After that, the expanded data are divided into training and testing sets of the ELM for analyzing and predicting the dyeing time of the nonwoven fabric. Compared with the back propagation (BP) neural network and the radial basis function (RBF) neural network, the results verify that the accuracy of the MCELM is highest. Furthermore, the relationship between the physical and structural properties of the nonwoven fiber material and the liquid penetration and capillary absorption capacity is established from a new perspective, which can provide production efficiency for nonwoven fiber.

The content of this paper will be organized in the following form: Section 2 introduces related research about the dyeing industry. Section 3 illustrates the ELM and MC in detail and puts forward the MCELM method for a better effect. In Section 4, a case study about analyzing and predicting in the dyeing industry is explored. Section 5, at the end of this paper, concludes the experimental result.

## 2. Related Work

With the occurrence of COVID-19, people’s demand for masks has increased, leading to extensive research on nonwoven fabric infection models of its raw materials. Horvath and Stanley [5] explored the infiltration process of fluid driven by capillary force in porous media through experiments; exploring the influence of the change of void structure on the seepage rate through mesoseepage simulation, they obtained the power function relationship between fluid rise height and time. Bijeljic et al. [6] used a topology network model to simulate the dynamic process of the capillary climb of the wetting liquid in the porous medium with accumulated particles under the weight field. Wiklund and Uesaka [7] used the LBM (Lattice Boltzmann Method) to investigate the capillary flow process of the liquid in fibrous porous media. Through numerical simulation results, they observed the phenomenon of unsaturated liquid flow in the pore structure. The above method explores the seepage process in porous media based on the mechanism model, but the modeling process of these methods is complicated and takes too long. Therefore, the simulation modeling of nonwoven fabric infection is carried out through a data-driven method. The modeling method has simple modeling and high accuracy. Nordlund and Lundstrom [8] used a finite element method for studying the effect of model geometric parameters on the permeability of fiber-stacked sewing fabrics (NCFs). The orderly and disorderly arrangement of fibers will affect permeability. Chen et al. [9] used the MC method to explore the flow of liquid in disorderly arranged porous media, and derived an empirical model related to permeability and fiber spacing. However, the traditional statistical data modeling method used to evaluate seepage results has the disadvantages of limited experimental samples and high cost.

Due to the disadvantages of conventional methods and the rapid development of artificial intelligence, many neural network methods are used in dyeing cloth and related industries. Srinidhi et al. put forward a Levenberg–Marquardt-based BP method to predict the dye solubility in supercritical carbon dioxide accurately, which overcame the problem in the dyeing industry [10]. Laura et al. proposed a system that can generate color formula when combined with the BP algorithm, which could control small changes in color and reduce the subjectivity of visual evaluation of the color of textile products [11]. Similarly, for the key issue of color matching in the dyeing industry, Shen et al. also combined the spectrophotometric color matching algorithm with the BP method to predict the color matching for top-dyed mélange yarn [12]. In addition to the BP, the RBF neural network has also been widely applied to prediction models. Schubert et al. applied the RBF and genetic algorithm (GA) to the oxidative decolorization of the stubborn dye, Reactive Black 5. Experiments showed that the combined method has a higher efficiency value [13]. Li et al. used the RBF and the GA to match the color for dyeing uniformly, which had the characteristics of costing less and predicting accurately [14]. Liu et al. presented an RBF based prediction model to improve spectral accuracy and characterization chromaticity [15]. Lu et al. put forward a novel RBF method using particle swarm optimization (PSO) to help match the computer color, which had an excellent performance in operation and time consumption [16]. However, the BP and the RBF are prone to local optimization and overfitting.

The ELM was proposed in 2004 to improve learning efficiency and simplify learning parameters [17]. Zhou et al. established a novel model combining ELM, differential evolution (DE), and a whale optimization algorithm (WOA) to categorize the chromatic aberration for increasing the classifying accuracy of print and dye solid color products [18]. Zhou et al. introduced the ELM on the basis of wavelet decomposition to effectively reflect the characteristics of textile defects and reduce the limitations of manual operation [19]. Chen et al. used the ELM method, combined with near-infrared (NIR) spectroscopy, to measure the composition of textiles, which helped to control their quality and save analysis time and cost [20].

Generally, sufficient and effective data can provide support and help for subsequent experiments. However, the amount of dyeing data obtained is small, which probably makes the error higher and robustness poor. Therefore, many virtual sample generation (VSG) methods for small samples were proposed by scholars to solve the above drawbacks. Li et al. put forward a mega-trend-diffusion method to generate samples more accurately [21]. According to an information-expanded function derived from triangular membership, Chen et al. developed a VSG method combining particle swarm optimization on the industrial processes, which obtained a better effect [22]. Yang et al. verified that the classifier using a Gaussian distribution based on the VSG is more effective [23]. However, the above methods require prior knowledge and acquired parameters for a precise result. Therefore, the MC method has been adopted by many scholars. Otsuki used the MC method for generating a matrix, which helped to analyze in turbid spherical samples [24]. Gong et al. presented a novel method, combined with the MC and the PSO, for generating virtual samples, which provided the data for the experiment of petrochemical processes [25]. Jérôme et al. used the MC method to find a high-performance working fluid with an uncertain property [26]. Guo et al. obtained, by the MC, a great deal of expanded typhoon data for analyzing wind hazards and optimizing wind field models [27]. Therefore, this paper proposes the ELM combined with the MC method to predict and analyze dyeing time with a small number of samples.

## 3. The Extreme Learning Machine (ELM) Combining the Monte Carlo (MC) Algorithm

### 3.1. The ELM

**Figure 1:**
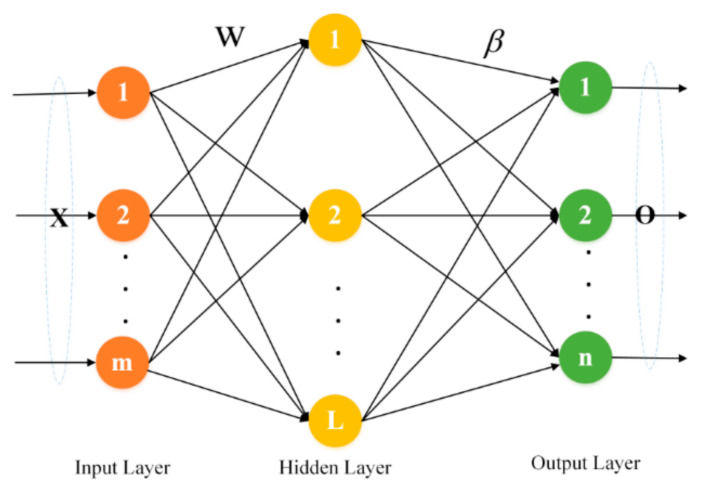
The network structure of the extreme earning machine (ELM).

For the ELM, the connection weight between the input and the hidden layer, and the threshold of the hidden layer, can be generated at random, without subsequent adjustment. The weight between the hidden and the output layers is directly calculated by mathematical methods and does not need adjusting iteratively. Therefore, the ELM has obvious advantages over other networks in terms of generalization performance and speed. The network structure of the ELM is shown in Figure 1.

Assume there are K arbitrary samples $\left\{\left(X_{k},Y_{k}\right)|k=1,2,\ldots ,K;X_{k}\in R^{m};Y_{k}\in R^{n}\right\}$. Among them, $X_{k}={\left[x_{k1},x_{k2},\ldots ,x_{km}\right]}^{T}$ represents the input of the ELM, correspondingly, $Y_{k}={\left[y_{k1},y_{k2},\ldots ,y_{kn}\right]}^{T}$ means the output. Meanwhile, suppose the neural network has only one hidden layer with the number of L. When the activation function is h(x), the neural network can be expressed as Equation (1).

(1) $$O_{k}=\overset{L}{\underset{l=1}{\sum }}\beta_{l}h\left(w_{l}\cdot X_{k}+v_{l}\right),k=1,2,\ldots ,K$$

where $w_{l}={\left[w_{l1},w_{l2},\ldots ,w_{lm}\right]}^{T}$ represents the weight between the input and the hidden layer node, $v_{l}$ is the threshold of the hidden layer node. On the whole, $w_{l}\cdot X_{k}$ is the inner product of $w_{l}$ and $X_{k}$. Simultaneously, $\beta_{l}={\left[\beta_{l1},\beta_{l2},\ldots ,\beta_{ln}\right]}^{T}$ represents the weight between the hidden and the output layer. Therefore, Equation (1) is rewritten as Equation (2).

(2) $$O=H\beta =\left[\begin{array}{cccc} h\left(w_{1}\cdot X_{1}+v_{1}\right) & h\left(w_{2}\cdot X_{1}+v_{2}\right) & \cdots & h\left(w_{L}\cdot X_{1}+v_{L}\right)\\ h\left(w_{1}\cdot X_{2}+v_{1}\right) & h\left(w_{2}\cdot X_{2}+v_{2}\right) & \cdots & h\left(w_{L}\cdot X_{2}+v_{L}\right)\\ \vdots & \vdots & \ddots & \vdots \\ h\left(w_{1}\cdot X_{K}+v_{1}\right) & h\left(w_{2}\cdot X_{K}+v_{2}\right) & \cdots & h\left(w_{L}\cdot X_{K}+v_{L}\right) \end{array}\right]\times \left[\begin{array}{c} \beta_{1}\\ \beta_{2}\\ \vdots \\ \beta_{L} \end{array}\right]$$

Among them, H represents the output of the hidden layer node, *β* means the output weight, and O is the output. $w_{l}$, $v_{l}$ and $\beta_{l}$ are expected to be special values $\hat{w_{l}}$, $\hat{v_{l}}$ and $\hat{\beta_{l}}$. At this time, the result of the neural network O can be close to the expected value Y, and the error is minimized. The objective function is min‖O−Y‖. Since the $w_{l}$, $v_{l}$ can be generated at random, the *H* can be uniquely determined. Therefore, training a single-layer neural network can be transformed into solving Equation (3).

(3) Hβ=Y

The solution of Equation (3) is shown in Equation (4).

(4) $$\hat{\beta }=H^{+}Y$$

where $H^{+}$ is the Moore–Penrose generalized inverse of *H*.

In summary, the process of the ELM algorithm is as below:

Step 1: Randomly generate the weight between the input and the hidden layer, as well as the hidden layer threshold, and determine the activation function.
Step 2: Calculate the hidden layer output $H=h\left(X\cdot w+v\right)$.
Step 3: Get $H^{+}$ and obtain the output layer weight $\hat{\beta }$ according to $\hat{\beta }=H^{+}Y$.

### 3.2. The MC Algorithm

The MC algorithm is a random simulation method based on probability and statistical theory. It aims to establish a probability model or random process whose parameters are equal to the solution of the problem, and then use a computer to complete a statistical simulation or sampling, calculate the statistical characteristics of the required parameters, and then obtain the approximate solution to the problem [28]. The reliability parameters of the system are expressed as Equation (5).

(5) $$R=\int_{\epsilon }G\left(A\right)$$

where $A=\left[a_{1},a_{2},\cdots ,a_{t}\right]$ represents the system state, $G\left(A\right)$ means the dependable function, and ϵ expresses the space for integral.

The MC algorithm has significant advantages. For example, it can effectively avoid the dimensionality problem because its convergence probability and speed are independent of the dimensionality. At the same time, it is unlimited on the distributions of events with high feasibility and can solve the problem of small samples well. In conclusion, the processes of the MC algorithm are as below:

Step 1: Describe the probability process.
Step 2: Sampling from a known probability distribution.
Step 3: Establish an estimate.

### 3.3. The Framework of ELM Combining the MC Algorithm

The proposed method expands the data samples through the MC algorithm to provide sufficient and reliable samples for subsequent experiment, and then realizes the prediction of the samples through the ELM method. The steps of the MCELM are as follows:

Step 1: Obtain the basic dataset for the experiment with the help of simulation software.
Step 2: Enhance sample size through the MC algorithm.
Step 3: Verify that there is no significant difference between the original data and the extended data by the hypothesis test.
Step 4: Use the samples obtained in Step2 to train the ELM and compare the output of the network with the real result.
Step 5: Obtain the MCELM model and analyze the prediction result according to the different error values.

**Figure 2:**
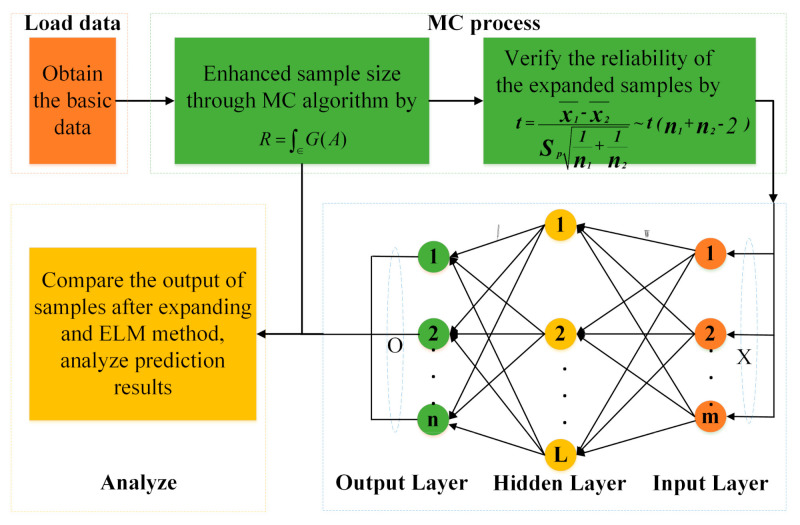
The flow of Monte Carlo integrating extreme learning machine (MCELM).

The flow of the MCELM method is shown in Figure 2 for a clearer description.

## 4. Case Study: Prediction of the Time Required of the Dyeing Process

**Figure 3:**
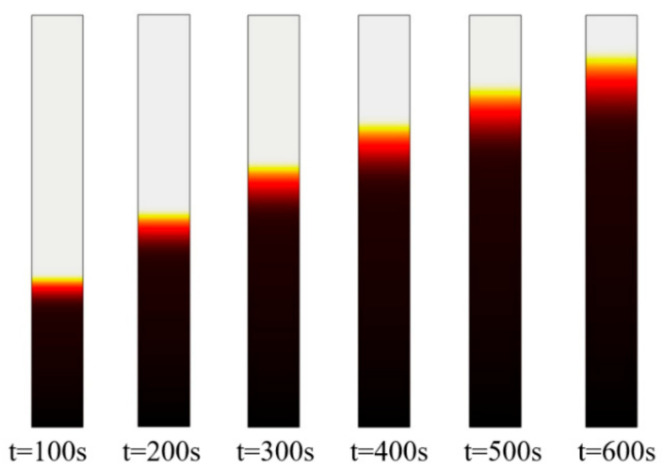
Effect diagram of the nonwoven fabric impregnation process (the above picture is from the first group, porosity 0.6-pore 4.75 × 10^−7^ m).

The dyeing industry is closely related to our daily lives, but the time required for dyeing is difficult to determine, leading to a certain amount of wasted time and resources. The proposed MCELM can predict the dyeing time well and bring greater reference value for related personnel. The multiphysics simulation software, COMSOL (COMSOL Inc., Stockholm, Sweden) can be used to numerically simulate the nonwoven fabric impregnation process, and the effect diagrams obtained at different times are as follows. Simulation requires complex model construction, parameter setting and complex calculation process. Figure 3 below shows the impregnation process of a nonwoven material with a porosity of 0.6 and a pore size of 4.75 × 10^−7^ m.

The specific parameter settings and formulas of the above picture are described in Table 1. The physical parameters, such as the density and viscosity of water and the density and viscosity of air, are set according to theoretical values. The pore size and porosity, and the geometric parameters of the nonwoven fabric, are set by actual needs, and the surface tension and permeability are calculated by the capillary model [29].

### 4.1. Data Analysis

**Figure 4:**
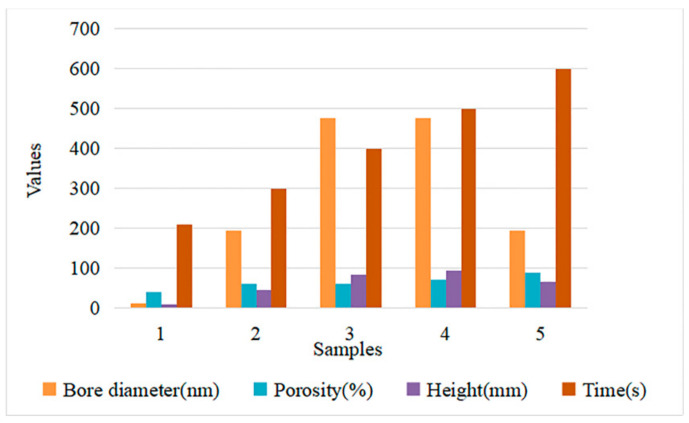
Sample values of real dyeing data.

In this experiment, a set of real dyeing data is collected, as shown in Figure 4. Among them, the bore diameter and height of the dyeing facility in the units of the nanometer (nm) and millimeter (mm), and the porosity (%), is used as the input variable of the ELM. Correspondingly, the dyeing time is the output of the neural network, whose unit is second (s). However, due to the small amount of real dyeing data, some data expansion methods, based on the MC, need to be used to supplement the sample size.

### 4.2. Prediction of the Time Required of the Dyeing Process

Since the data used in the ELM are generated by the MC algorithm, the extended data should be checked first, whether there exists a significant difference from the original data. For the above purpose, hypothesis testing is performed before analyzing and predicting the dyeing time.

#### 4.2.1. Verification of Data Samples Obtained by the MC Method

In order to prove that the expanded data can be applied to the input of the neural network, it needs to be tested to show it is not significantly different from the original data. The *T*-test in this paper is used to infer the significant difference between the mean of the sample population through sample data. First, the null hypothesis $H_{0}$ is established, which means that the original data and the expanded data are not significantly different. Next, whether the variances of the two populations are equal needs to be judged. The Levene F test is used here. If the *P*-value corresponding to the *F*-value is greater than the significance level, it is considered that there is no significant difference in the variances of the two populations. At this time, the t statistic can be constructed as Equation (6).

(6) $$t=\frac{\bar{x_{1}}-\bar{x_{2}}}{S_{p}\sqrt{\frac{1}{n_{1}}+\frac{1}{n_{2}}}}∼t\left(n_{1}+n_{2}-2\right)$$

Among them, $n_{1}$ means the sample size of the original data, while $n_{2}$ expresses the sample size of the expanded data. At the same time, $\bar{x_{1}}$ and $\bar{x_{2}}$ represent the mean of the two samples, respectively. $S_{p}$ represents the combined standard deviation, and its calculation method is shown in Equation (7).

(7) $$S_{p}=\sqrt{\frac{\left(n_{1}-1\right)S_{1}{}^{2}+\left(n_{2}-1\right)S_{2}{}^{2}}{n_{1}+n_{2}-2}}$$

where $S_{1}$ and $S_{2}$ mean the standard deviations of the two samples, separately. After the *T*-value is obtained, it can be determined whether the null hypothesis can be accepted under a certain confidence level, according to the degree of freedom. If the *P*-value is greater than the significance level α, the null hypothesis is accepted; that is, there is no significant difference between the two population means. The group statistics result is described in Table 2.

The independent sample test results are shown in Table 3, where E_va_ expresses equal variances assumed, and E_vna_ means E_qual_ variances not assumed.

**Figure 5:**
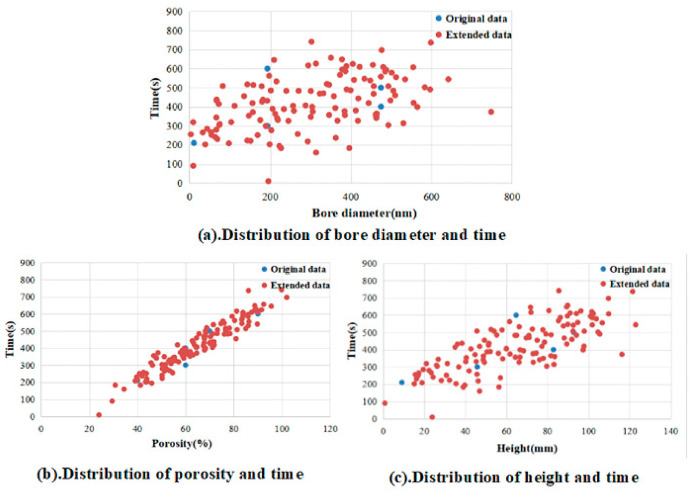
Comparison of the distribution of original data and extended data. (Among which, subfigures (**a**–**c**) represent the corresponding relationship between bore diameter, porosity, height and time, respectively).

Taking the input variable bore diameter as an example, from Table 3, the variances of the two populations can be judged to be equal. Because, in Levene’s test, the *F*-value is 0.36, which is greater than 0.05, the $H_{0}$ of equal variances cannot be rejected; that is, the *T*-test under the condition of equal variance can be selected. At this time, *t* = −0.427, the corresponding probability *P*-value is 0.67, which is greater than 0.05, so, the $H_{0}$ is accepted; namely there is no significant difference between the two samples. Similar analyses of the remaining variables show that there is no significant difference between the original data and the expanded data. In conclusion, it is feasible to expand the data through the MC algorithm. The comparison between the original data and the expanded data is shown in Figure 5.

#### 4.2.2. The Time Required Analysis and Prediction of the Dyeing Process

**Figure 6:**
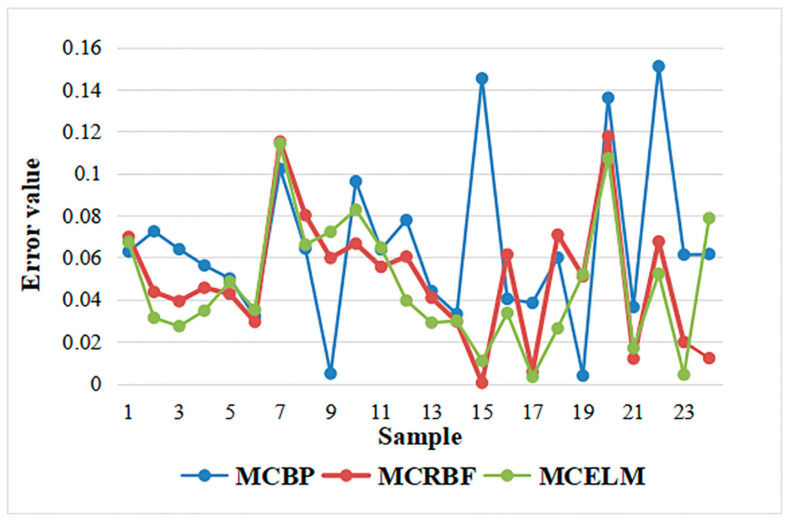
Prediction error comparison of MCBP, MCRBF, MCELM with 15 nodes.

Based on the above data samples, the BP network combined with the MC algorithm (MCBP), the RBF combined with the MC algorithm (MCRBF), and the MCELM are, respectively, used to predict the time in the dyeing process. First, the hidden layer node is set to 15. At this time, the errors of the MCBP, the MCRBF, and the MCELM can be seen as Figure 6.

**Figure 7:**
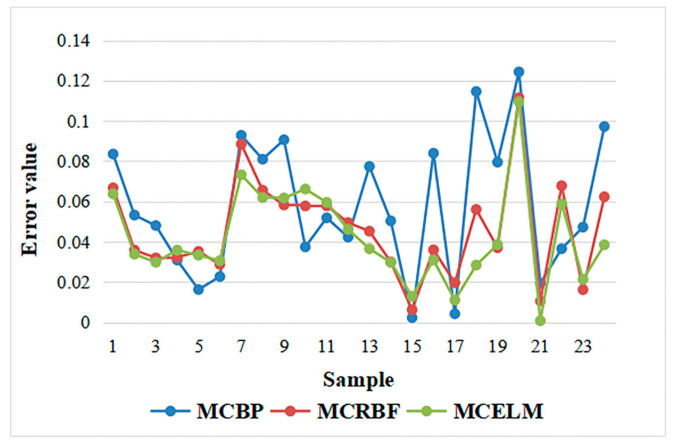
Prediction error comparison of the MCBP, the MCRBF, and the MCELM with 10 nodes.

At this time, the prediction effect is poor with many errors exceeding 0.1. Adjust the number of hidden layer nodes of the neural network to 10. The errors of the MCBP, the MCRBF, and the MCELM are described in Figure 7.

**Figure 8:**
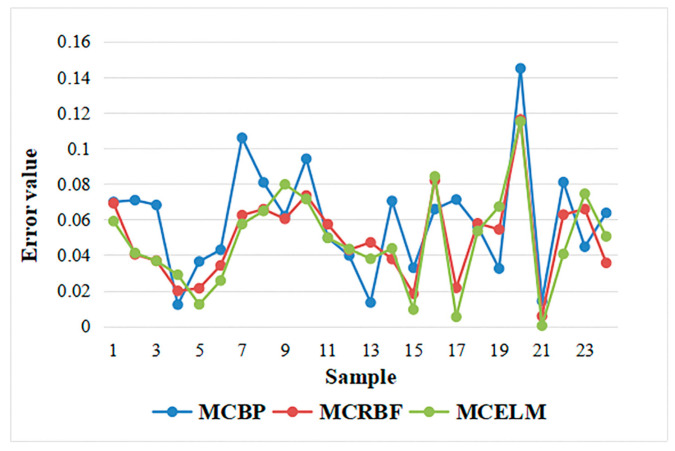
Prediction error comparison of the MCBP, the MCRBF, and the MCELM with 5 nodes.

It can be seen from Figure 7 that, when the number of hidden layer nodes is 10, each model performs better than 15 hidden layer nodes and is relatively stable. Finally, reduce the number of hidden layer nodes to 5, and the error prediction comparison chart is shown as Figure 8.

Figure 8 shows that the prediction errors of the MCBP, the MCRBF, and the MCELM models are also relatively low, in this case, but are higher than the error when the number of nodes is 10. In order to more clearly describe the errors when the number of hidden layer nodes are 15, 10, and 5, the overall comparison is described in Table 4.

**Figure 9:**
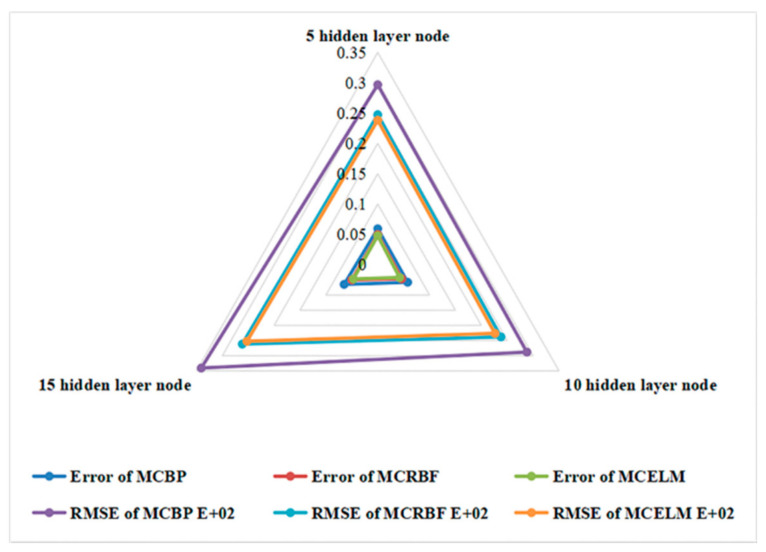
Comparisons of overall Error and root mean squared error (RMSE).

At the same time, Root Mean Squared Error (RMSE) is also shown in Figure 9. It can be seen that the overall error and the RMSE of the MCELM are lower than the MCBP and the MCRBF. In addition, when the number of hidden layer nodes is 10, the MCELM has the lowest error of 4.22%, which is 1.56% and 0.39% lower than the MCBP and the MCRBF, respectively. Therefore, the time prediction for the dyeing process is the most accurate.

**Figure 10:**
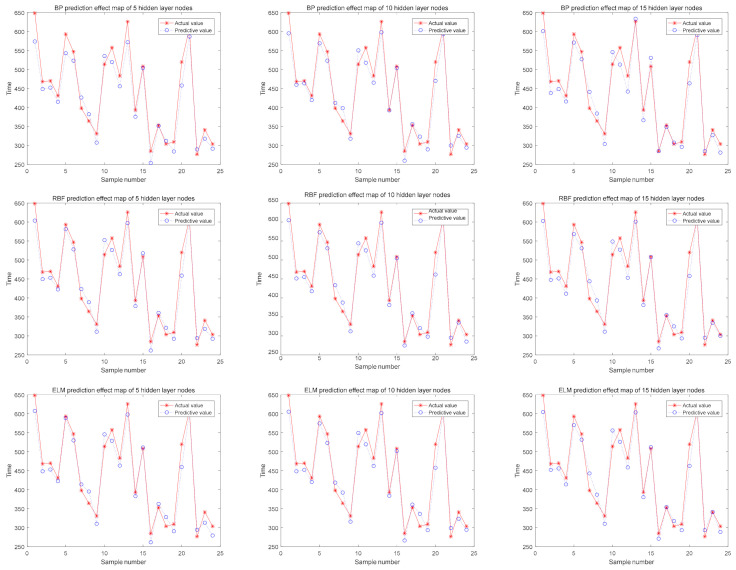
Prediction time comparison of different algorithms.

The comparison between the actual value and predictive value is shown in Figure 10. It is easy to see that the predictive value of the RBF and ELM methods are closer to the actual value. With the analysis of MRE and RMSE, the ELM method performs best.

Based on the above data samples, each datum usually takes 90s to run with COMSOL each time. At the same time, the MCBP, the MCRBF, and the MCELM models do not exceed 6s, especially the MCELM, which can realize the prediction of the immersion time simply and quickly. The overall run time is shown in Table 5.

Precisely predicting the speed and time of nonwoven material impregnation through the proposed method is of great significance for improving the quality and yields of nonwoven materials, which can meet raw material requirements for making masks with the global spread of COVID-19. In the field of sanitary products, and with the development of economies, people have higher and higher quality requirements for absorbent sanitary products, such as sanitary pads and baby diapers, and requirements for water absorption and the penetration of materials are also higher and higher. With the global spread of the novel coronavirus, the demand for disposable protective equipment, such as masks, has also soared. How to protect individuals from the virus, while keeping the skin dry and breathable, also generates higher requirements from nonwoven materials. In addition, in biochemical medicine, the application fields of nonwoven materials, such as testing and building materials, have different requirements from the permeability and water absorption of nonwoven fabrics. Precisely predicting the speed and time of nonwoven material impregnation through machine learning is of great significance for improving the quality of nonwoven materials and products.

## 5. Conclusions

In this paper, we propose an improved MCELM method for predicting the time of nonwoven fabric dip. The MC method is applied to expand the sample size of nonwoven fabric simulation data. There is no significant difference between the data samples expanded by the MC method and the original data in terms of the *T*-test. Then, the ELM is used to establish a prediction model between nonwoven fabric soaking time and physical properties. Compared with the predictions of several different algorithms with different numbers of hidden error layers, the minimum error of the MCELM is 4.22%, which is 1.56% and 0.39% lower than the MCBP and the MCRBF, respectively. In addition, the prediction time of the proposed method is greatly reduced, and the soaking time of nonwoven fabrics can be given in a short time based on the physical properties of nonwoven fabrics, which has obvious advantages over traditional modeling analysis and simulations. Since nonwoven fabric disease detection has been widely used, the detection effect is related to the penetration of liquid in the nonwoven fabric material, and it can accurately and quickly predict the percolation speed of a certain liquid in the nonwoven fabric. The detection effect, accuracy, and sensitivity of the bucky microfluidic chip have a direct impact. Furthermore, the proposed method can quickly and accurately predict the soaking time of nonwoven fabrics, which is significant for applying nonwoven fabrics to different fields.

## Figures and Tables

**Table 1:** Parameter setting.

Physical Parameters	Expression	Value	Unit
Density of water	rho-water	1 × 10^3^	kg/m^3^
Water viscosity	mu-water	2.98 × 10^−3^	Pa·s
Surface Tension	gamma	0.0723	N/m
Density of air	rho-air	1.293	kg/m^3^
Air viscosity	mu-air	1.79 × 10^−5^	Pa·s
Porosity	por	0.6	—
Bore diameter	R_c_	4.75 × 10^−7^	m
Permeability	$k=\frac{por}{8}\times R_{c}^{2}$	1.69932 × 10^−10^	m^2^
Contact angle	theta	0	°
Inlet capillary pressure	$pec=2\cdot gamma\cdot \cos\;\left(theta\right)/R_{c}$	3.044 × 10^−7^	Pa
length	L_0_	12	cm
width	W_0_	1.5	cm
height	th	1	mm

**Table 2:** Group statistics.

	Group	Mean	Standard Deviation	Standard Error Mean
Bore diameter(nm)	Original data	269.36	201.922	90.302
	Expanded data	301.905	165.946	14.902
Porosity (%)	Original data	64	18.166	8.124
	Expanded data	65.011	16.316	1.465
Height(mm)	Original data	59.008	33.187	14.842
	Expanded data	64.211	27.894	2.505
Time(s)	Original data	402	154.984	69.311
	Expanded data	417.4	141.248	12.684

**Table 3:** Independent-samples *T*-test result.

		Levene’s Test for Equality of Variances		*T*-Test for Equality of Means				
		F	Significance	t	Degrees of Freedom	Significance (Two-Tailed)	95% Confidence Interval of the Difference	
							Lower	Upper
Bore diameter (nm)	E_va_	0.36	0.55	−0.427	127	0.67	−183.461	118.371
	E_vna_			−0.356	4.221	0.739	−281.5	216.41
Porosity (%)	E_va_	0.025	0.874	−0.014	127	0.893	−15.793	13.772
	E_vna_			−0.122	4.264	0.908	−23.381	21.36
Height (mm)	E_va_	0.063	0.802	−0.406	127	0.685	−30.544	20.139
	E_vna_			−0.346	4.231	0.746	−46.108	35.703
Time (s)	E_va_	0.007	0.934	−0.238	127	0.812	−143.303	112.502
	E_vna_			−0.219	4.272	0.837	−206.214	175.413

**Table 4:** Comparison of overall errors.

	5 Hidden Layer Node		10 Hidden Layer Node		15 Hidden Layer Node	
	MRE	RMSE	MRE	RMSE	MRE	RMSE
MCBP	0.0593	29.657	0.0578	28.805	0.0649	34.069
MCRBF	0.0495	24.719	0.0461	23.793	0.0498	26.157
MCELM	0.048	23.802	0.0422	22.630	0.047	25.246

**Table 5:** Comparison of running times of different algorithms.

	BP	RBF	ELM
5 hidden layer node	4.821386 s	4.128717 s	3.398797 s
10 hidden layer node	4.901459 s	4.031216 s	3.449390 s
15 hidden layer node	5.045841 s	4.521651 s	3.348385 s

## Data Availability

The data presented in this study are available on request from the corresponding author.

## Abbreviations

ELM	Extreme learning machine
MC	Monte Carlo
MCELM	Extreme learning machine combing Monte Carlo
*T*-test	Student’s *t* test
BP	Back propagation
RBF	Radial basis function
GA	Genetic algorithm
PSO	Particle swarm optimization
DE	Differential evolution
WOA	Whale optimization algorithm
NIR	Near-infrared
VSG	Virtual sample generation
SLFNs	Single hidden layer feed-forward neural networks
$X_{k}$	The input of ELM
$Y_{k}$	The output attributes of samples
*L*	Number of hidden layer nodes
*h*(*x*)	The activation function
$O_{k}$	The output of ELM
$w_{l}$	Weight between the input layer node and the hidden layer node
$\beta_{l}$	Weight between the hidden layer and the output layer
$v_{l}$	Threshold of the hidden layer node
*H*	Hidden layer output matrix
$H^{+}$	Moore-Penrose generalized inverse
$G\left(A\right)$	Dependable function
nm	Nanometer
mm	Millimeter
s	Second
Eva	Equal variances assumed
Evna	Equal variances not assumed
MCBP	BP network combined with MC algorithm
MCRBF	RBF combined with MC algorithm
RMSE	Root mean squared error
